# Thymol alleviates silica dioxide nanoparticle-induced reproductive performance toxicity via antioxidant and anti-inflammatory mechanisms in male rats

**DOI:** 10.1038/s41598-025-07769-x

**Published:** 2025-07-04

**Authors:** Mahmoud A. Khedr, Amira A. Goma, Rashed R. Rashed, Hossam G. Tohamy, Mustafa Shukry, Sara E. El-Kazaz

**Affiliations:** 1 Department of Animal Husbandry and Animal Wealth Development, Faculty of Veterinary Medicine, Alexandria University, Alexandria, 21944 Egypt; 2 Department of Pathology, Faculty of Veterinary Medicine, Alexandria University, Alexandria, 21944 Egypt; 3 Department of Physiology, Faculty of Veterinary Medicine, Kafrelsheikh University, Kafrelsheikh, 33511 Egypt

**Keywords:** Silica dioxide nanoparticles, Thymol, Sexual behavior, Gene expression, Oxidative stress, Steroidogenesis, Spermatogenesis, Physiology, Natural hazards

## Abstract

Nanoparticles (NPs) have gained increasing attention due to their unique physicochemical properties and broad applications. However, concerns about their potential toxicity, particularly reproductive toxicity, have emerged. Silica dioxide nanoparticles (SiO₂-NPs) are among the most commonly used NPs and have been linked to adverse effects on male reproductive health. This study aimed to evaluate the potential ameliorative effect of thymol, a natural monoterpene phenol with antioxidant and anti-inflammatory properties, against SiO₂-NPs-induced reproductive toxicity in male rats. Twenty-four adult male Sprague-Dawley rats were randomly assigned to four groups: control, SiO₂-NPs-treated (10 mg/kg body weight, intraperitoneally), thymol-treated (30 mg/kg body weight, orally), and SiO₂-NPs + thymol co-treated. Treatments were administered daily for 56 days. Male reproductive performance was evaluated through sexual behavior assessment, sperm characteristics, reproductive hormone levels, oxidative stress markers, inflammatory biomarkers, gene expression analysis, and histopathological examination of testicular tissue. Results revealed that SiO₂-NPs significantly impaired reproductive performance, indicated by reduced sperm motility, viability, and count, along with increased sperm abnormalities. Thymol co-administration significantly restored testosterone levels and partially normalized elevated LH and FSH levels caused by SiO₂-NPs, indicating endocrine protection. Moreover, SiO₂-NPs induced oxidative stress, elevated pro-inflammatory cytokines (TNF-α and IL-6), and disrupted the expression of key genes associated with oxidative stress response (NRF2), apoptosis (BAX, BCL-2), steroidogenesis (STAR, CYP11A1), and spermatogenesis (PRM1, GATA4). Thymol co-administration with SiO₂-NPs significantly mitigated these adverse effects by restoring antioxidant levels, reducing inflammation, and improving gene expression and the histological architecture of the testes. The findings suggest that thymol has a promising protective role against SiO₂-NPs-induced male reproductive toxicity through its antioxidant and anti-inflammatory actions.

**Supplementary Information:**

The online version contains supplementary material available at 10.1038/s41598-025-07769-x.

## Introduction

Nanoparticles (NPs) have gained more interest in the last few decades due to their exceptional characteristics, including size, surface area, optical features, and biocompatibility^1^. One of the most widely used nanomaterials across various industries is Silica nanoparticles (Si-NPs). For example, in the construction field, consumer products, electronics, biomedicine, and pharmaceutical products are used^2^. This is because of their unique physico-chemical properties.

The toxicological effects of nanoparticles have become a growing concern, especially in relation to reproductive health. The reproductive system is particularly vulnerable to external stressors due to its role in transmitting genetic material^3^. Silica dioxide nanoparticles (SiO₂-NPs) are extensively used in biomedical and industrial applications, but growing evidence highlights their potential reproductive toxicity^4^. The risks associated with SiO₂-NPs on the reproductive system are receiving increased attention^5^. SiO₂-NPs were observed to produce sperm deformity and decrease the number of sperm and their motility in male rats^6^. It also harms Sertoli cells^7^ and causes a reduction in Leydig cell count of rats^8^. Moreover, it was reported to affect sperm maturation in the epididymis, causing decreased sperm count and quality and energy metabolism dysfunction in mice^9^. SiO₂-NPs can penetrate the blood-testis barrier, accumulate in testicular tissue, and induce oxidative stress through excessive ROS generation, leading to lipid peroxidation and DNA damage^9^. This oxidative damage disrupts spermatogenesis, reduces sperm count and motility, and increases abnormalities.

Additionally, SiO₂-NPs impair mitochondrial function and downregulate key genes involved in steroidogenesis (*STAR*, *CYP11A1*) and spermatogenesis (*PRM1*, *GATA4*), resulting in hormonal imbalance and germ cell apoptosis^10–12^. Histologically, they cause degenerative changes in seminiferous tubules and interstitial edema^13^. Due to the reported harmful impact of Silica nanoparticles on male reproductive function, identifying an effective agent that could ameliorate and/or mitigate their adverse effects on male reproductive health become a more critical issue. Recent studies have highlighted growing interest in natural antioxidants like thymol for their therapeutic potential^14^. Dietary phytochemicals like thymol have been in the spotlight recently due to their promising pharmacological, physicochemical, and pharmacokinetic properties^14^. An important component of thyme essential oil and a wide variety of plants, such as Thymus vulgaris, Nigella sativa, Thymus ciliates, and Origanum vulgarae, is thymol, a monoterpene phenolic chemical^14^. Thymol displays a wide range of biological functions, including antioxidant^15^ and anti-inflammatory properties^16^. Thymol’s antioxidant properties have been demonstrated in various models, including Chinese hamster fibroblast cells (V79)^17^, intestinal Caco-2 cell line^18^, and rodent models exposed to oxidative stressors such as titanium dioxide and methomyl^19^. The phenolic hydroxyl group, in its structure, could scavenge and neutralize free radicals^14^. Moreover, previous studies reported thymol’s ability to improve sperm quality evaluations^20^.

Güvenç et al.^20^ showed that thymol orally administered at dosages of (10 and 20 mg/kg BW) over 10 weeks in rats resulted in enhanced sperm concentration, motility, and viability while concurrently reducing sperm abnormalities in comparison to the control group. Moreover, Jafari et al.^19^ revealed that oral co-treatment of TiO2 nanoparticle-intoxicated rats with thymol (10 and 30 mg/kg BW/day) for 60 days increased the testicle weight in a dose-dependent manner compared to the group treated with TiO2 nanoparticles alone. In addition, thymol improved all sperm parameter abnormalities.

Accordingly, the current investigation set out to assess the potential ameliorative impact of Thymol against Silica dioxide nanoparticles (SiO2 NPs) induced reproductive toxicity in male rats through evaluation of sexual behavior, semen characteristics, reproductive hormones level, in addition to antioxidants, inflammatory markers and gene expression in testes, together with the testicular tissue histopathology.

## Materials and methods

### Chemicals

Silicon dioxide nanoparticles (SiO₂-NPs) were sourced from Sigma-Aldrich Co. (St. Louis, MO, USA), while Thymol was procured from Oxford Lab Fine Chem Co. (India).

### SiO2- NPs characterization

SiO₂ nanoparticles (SiO₂-NPs) were characterized at the Nanotechnology Center, Chemistry Department, Faculty of Science, Kafrelsheikh University, Egypt, using scanning electron microscopy (SEM), X-ray diffraction (XRD), and a zeta potential/particle size analyzer. The XRD analysis was carried out with a Shimadzu 6000–XRD diffractometer, employing Cu Kα radiation (λ = 1.54056 Å). The particle size and surface morphology were assessed using a JEOL (JSM-IT100) scanning electron microscope operating at 30 kV. Zeta potential values were determined using a Brookhaven zeta potential/particle size analyzer^21^.

### Ethical statement

All animal procedures and experimental protocols were conducted in accordance with the ARRIVE 2.0 guidelines for reporting in vivo experiments^22^. Ethical approval was granted by the Ethics Committee of Alexandria University Institutional Animal Care and Use Committee (ALEXU-IACUC, approval number 120/2022). All procedures were performed in compliance with the relevant institutional and international guidelines for the care and use of laboratory animals.

### Animals

Forty-eight (Twenty-four male and twenty-four female) Sprague-Dawley adult rats, aged 3–4 months, with an average body weight ranging from 160 to 180 g, were sourced from the Medical Research Institute, Alexandria University, Egypt. The rats were housed at the animal facility of the Medical Research Institute and the Medical Technology Center for Research and Services, Alexandria University, Egypt, and maintained under a natural light/dark cycle. They were given unrestricted access to food and water. The diet provided was a commercial broiler starter (Al-Eman Co., Egypt) containing 21% crude protein, 4.11% fat, and 2.44% crude fiber, aligning with the NRC dietary recommendations^23^. Prior to the initiation of the treatment, all rats were acclimated for two weeks.

### Experimental design

Twenty-four male rats were randomly assigned into four groups (6 each): (1) The control group received intraperitoneal injections of saline and oral administration of corn oil to account for the potential effects of both administration routes; (2) SiO2 -NPs treated group administered SiO2- NPs (10 mg/kg bwt) IP; (3) Thymol treated group received thymol (30 mg/kg bwt) orally via gavage, The selected dose of thymol (30 mg/kg bwt) was based on previously published studies demonstrating its efficacy and safety in rodent models without inducing systemic or reproductive toxicity^19,24,25^. Therefore, this dose was considered appropriate for assessing its protective role in the current study. The oral LD50 values in rats for thymol were 980 mg/kg body weight^26^ ; (4) SiO2 -NPs and Thymol treated group received both drugs. SiO2 -NPs were dispersed in normal saline and then sonicated for 5 min before use. Thymol was dissolved in corn oil. The treatment was administered daily for 56 days^24,27^; this experiment continued for 56 days to complete spermatogenesis and sperm maturation in the epididymis^28^. The doses and route of administration used for both drugs were according to^24,27^. The co-administration in the first and fourth groups was separated by time interval (30 min) with the start with the IP injection followed by the oral one. At the end of the treatment period, sexual behavior was determined by a fertility test. Afterward, rats were humanely euthanized by decapitation. Blood samples were collected for reproductive hormone level determination. Also, sperm characteristics and reproductive organ weights were evaluated. After organ weighting, one testicle was kept at − 80 ◦C for antioxidants, inflammatory biomarkers, and gene expression, whereas the second testicle was submerged in formalin solutions for histopathological analysis.

### Sexual behavior (fertility test)

Twenty-four female rats received intraperitoneal injections of Lutalyse^®^ (dinoprost tromethamine) at a dose of 0.1 mg/100 g body weight^29^ twice daily to synchronize the estrous cycle. Vaginal smears were taken to confirm estrous. Females in estrous were paired with males for mating within a plastic enclosure and documented on video for 15 min^30^. The following parameters were determined: mount frequency: mounts number till ejaculation; intromission frequency: intromissions number till ejaculation; mount latency: time from female entrance till the first mount; intromission latency: time from female entrance till the first intromission and ejaculatory latency: time from first intromission till ejaculation^31^.

### Reproductive hormones and reproductive organ’s weight

The rats were anesthetized with sodium pentobarbital (60 mg/kg; Sigma-Aldrich Co., St. Louis, MO, USA) and subsequently euthanized by decapitation. Blood samples were collected through cardiac puncture into tubes without anticoagulants. The samples were left to clot at 4 °C and then centrifuged at 3000 rpm for 10 min to separate the serum. The obtained serum was stored at -20 °C until it was used for analysis. Serum levels of testosterone, follicle-stimulating hormone (FSH), and luteinizing hormone (LH) were measured using rat-specific ELISA kits (Cusabio Biotech Co., Wuhan, China; Catalog Nos. CSB-E06869r for testosterone, CSB-E12654r for FSH, and CSB-E05100r for LH) following^24^. The sensitivity of the kits was 0.1 ng/mL for testosterone, 0.1 mIU/mL for FSH, and 0.05 mIU/mL for LH. Testicles, epididymis, prostate, and seminal vesicles were harvested from beheaded rats and weighed^32,33^.

### Sperm characteristics

The Sperm Characteristics were performed as follows, according to^34,35^. Epididymal sperm were collected by slicing the epididymis in a sterile petri dish, allowing sperm to be released from the tubules. A drop of the suspension was placed on a clean slide, covered with a cover slip, and examined under a light microscope at 400x magnification. The motility percentage was assessed within 2–4 min by observing at least 10 fields and calculating the proportion of motile sperm. For viability analysis, an equal drop of 1% eosin Y and 5% nigrosine stain was mixed with an equal drop of epididymal content, incubated for 2 min at room temperature, and examined at 400x magnification. Live sperm remained colorless, while dead sperm appeared dark pink, with viability determined by counting 100 sperm per slide. Sperm abnormalities were assessed by preparing a smear from a mixture of the stain and epididymal content, observing 100 sperm cells per slide at 400x magnification, and recording any head or tail deformities. For sperm count, 5 µl of epididymal suspension was diluted with 95 µl of a solution containing 5 g NaCl and five drops of formalin in 100 ml distilled water. A drop of the diluted sample was placed on a hemocytometer coverslip and left in a moist chamber for five minutes for sedimentation. Sperm cells were counted under a light microscope at 400x magnification.

### Antioxidants, oxidative stress, and inflammatory biomarkers

The testicular homogenate was centrifuged at 3000 rpm for 10 min at 4 °C^36^, after which it was utilized to extract testicular tissue from phosphate-buffered saline (PBS). The supernatant was collected for biomarker analysis. The assessed inflammatory markers included tumor necrosis factor-α (TNF-α) and interleukin-6 (IL-6)^36^, antioxidants including glutathione (GSH), catalase (CAT), superoxide dismutase (SOD), and malondialdehyde (MDA)^37,38^.

### Histopathological examination

Testicular tissue was collected from all treated groups and instantly fixed in 10% formalin for at least 24 h for histological analysis by light microscopy. Specimens were dehydrated in ascending concentrations of alcohol, cleared in xylene, and embedded in paraffin wax. . Furthermore, the grading of testicular lesions was described as follows: negative (−), very mild (+), mild (++), mild to moderate (+++), moderate (++++), and severe (+++++).

### RT-PCR

The gene expression in the testicles was assessed using quantitative real-time polymerase chain reaction. Total RNA was extracted from about 100 mg of testicular tissue using TRIZOL Reagents (Invitrogen, Carlsbad, CA, USA). A Nanodrop spectrophotometer was used to measure the quantities of RNA. A cDNA synthesis kit (Fermentas, Waltham, MA, USA) was used for complementary DNA (cDNA) synthesis, with only RNA samples showing an A260/A280 ratio of 1.8 or above being deemed appropriate. Table 1 shows the particular primers and SYBR Green Master Mix that were used to amplify the cDNA that was obtained. An internal reference gene used to normalize the expression levels was the glyceraldehyde 3-phosphate dehydrogenase (GAPDH) gene. In order to find the relative quantification of gene expression, the 2^(-ΔΔCt) calculation method was employed^39^.

### Statistical analysis

The Statistical Package for the Social Sciences (SPSS, version 25) was used for all statistical analyses. To explore relationships among multiple measured parameters (e.g., sexual behavior, sperm characteristics, and reproductive organ weights), Principal Component Analysis (PCA) was employed as an exploratory multivariate technique. PCA reduced the dimensionality of correlated variables by transforming them into a smaller set of uncorrelated variables—principal components—that retained most of the original variance. This method allowed for a clearer interpretation of interrelationships among outcomes while minimizing multicollinearity. PCA was not used for inferential hypothesis testing but rather to summarize data structure and guide subsequent analyses. Components with eigenvalues greater than 1 were retained, and variables with loadings greater than 0.60 were considered significant contributors.

To assess treatment effects on measured parameters, a one-way General Linear Model (GLM) was used. Data were presented as means ± standard error (SEM), and p-values < 0.05 were considered statistically significant. Post hoc comparisons between treatment groups were conducted using Duncan’s multiple range test to identify significant differences.

## Results

### Characterization of the SiO2 NPs

**Fig. 1:**
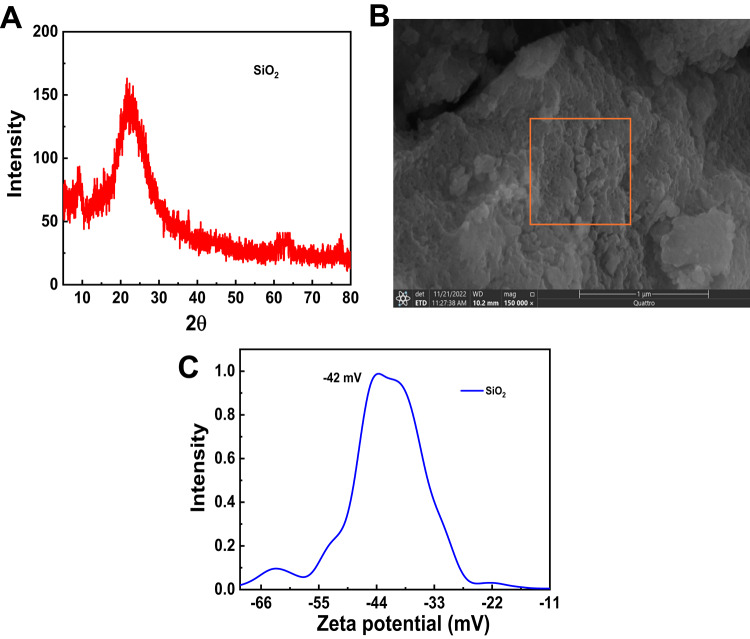
(A) X-ray diffractometer, (B) scanning electron microscopy (SEM), and (C) zeta potential/particle size showed that the average diameter of SiO₂-NPs was ˂ 50 nm. The rectangular shape showed groups of SiO₂-NPs.

Scanning electron microscopy (SEM), X-ray diffractometer, and zeta potential/particle size analysis showed that the average diameter of SiO2 NPs was < 50 nm (Fig. 1).

### Sexual behavior

The association between the five sexual behavior items of adult male rats (mount frequency, intromission frequency, mount latency, intromission latency, and ejaculatory latency) was tested using Principal component analysis. Two components had an eigenvalue greater than 1, explaining 79.82% of the total variance. After observing component coefficients, one item (ejaculatory latency) was removed as having a coefficient less than 0.60, and four items were retained in the two components, as shown in Table 2. Component 1 contains mount latency, intromission latency, and intromission frequency, whereas mount frequency comes in component 2.

**Fig. 2:**
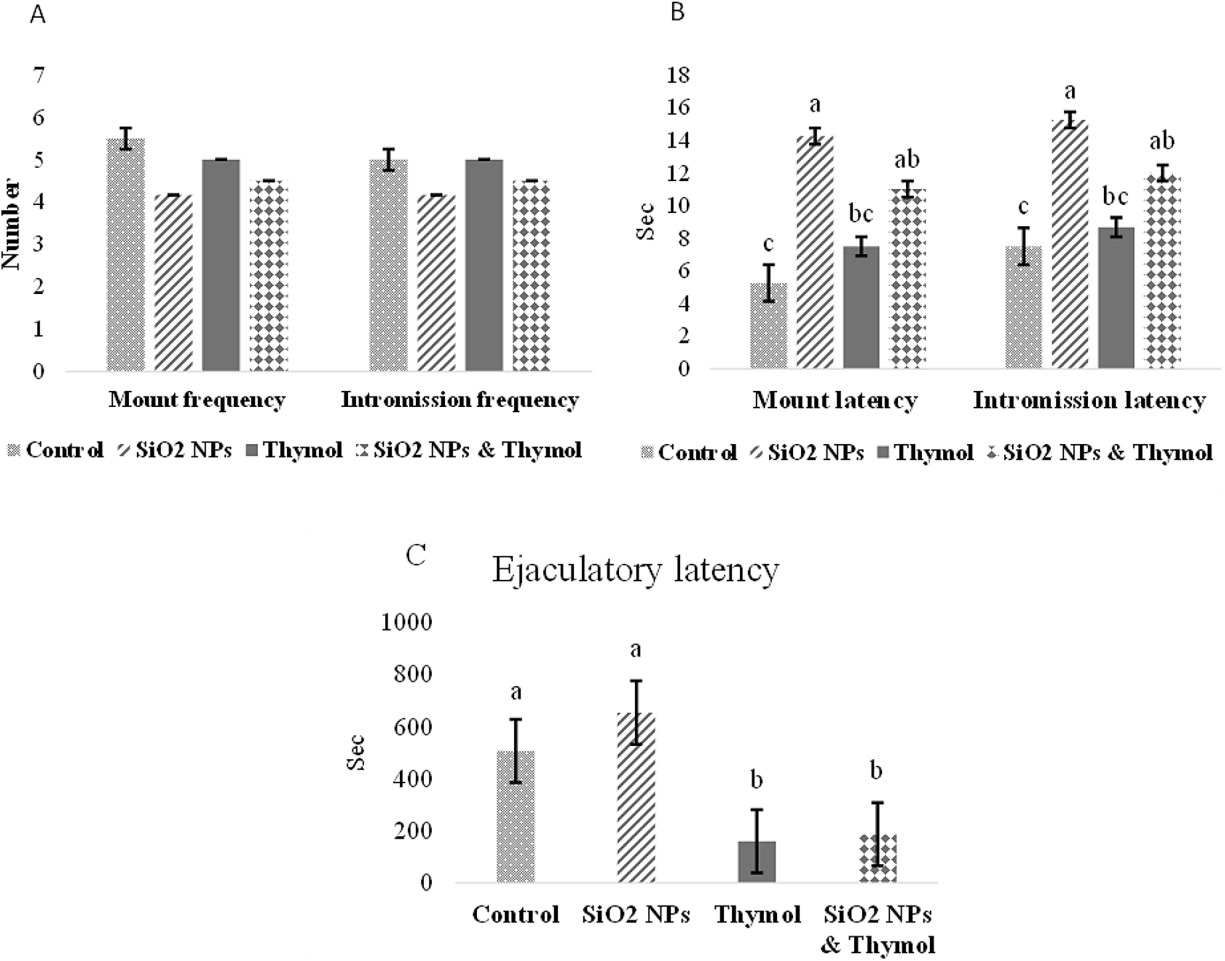
Effect of SiO₂-NPs and thymol on adult male rats’ sexual behavior during fertility test: (A) mount and intromission frequencies; (B) mount and intromission latencies (seconds); and (C) ejaculatory latency (seconds). All the values are expressed as the mean ± SEM. Different small letters(a-c) indicate significance at *p* < 0.05.

Figure 2A showed a non-significant difference between groups for the mount and intromission frequencies. However, there was a noteworthy increase in mount and intromission latencies in rats administered SiO2-NPs compared to control rats (Fig. 2B). Moreover, longer mount and intromission latencies were found in rats administered SiO2-NPs plus thymol than the control group, but this was shorter than SiO2-NPs treated group, although non-significant. Figure 2C, ejaculatory latency demonstrated a notable reduction in the rats that received SiO2- NPs plus thymol compared to the control and SiO2- NPs treated group.

### Reproductive organs weight

**Fig. 3:**
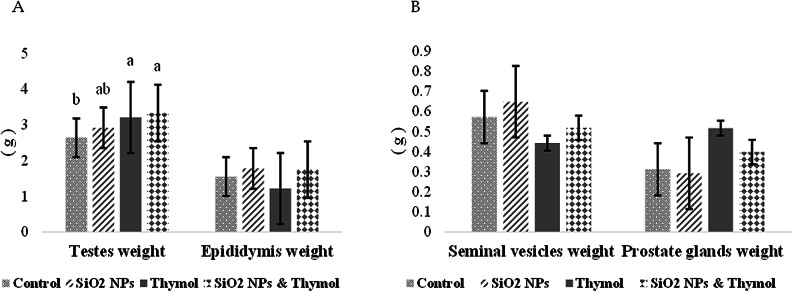
Effect of SiO₂-NPsand thymol on reproductive organs weight of adult male rats: (A) testicles and epididymis weights (gram); (B) seminal vesicles and prostate glands weights (gram). All the values are expressed as the mean ± SEM. Different small letters(a-b) indicate significance at *p* < 0.05.

The association between the four reproductive organs’ absolute weight (Testicles, epididymis, seminal vesicles, and prostate glands) of adult male rats was examined using principal component analysis. Two components had an eigenvalue greater than 1, explaining 72.52% of the total variance. After observing the components matrix, the two components with the four items were retained because they had coefficients > 0.60, as shown in Table 3. Testicles and epididymis weight come in one component, whereas seminal vesicles and prostate glands come in separate components. Figure 3A revealed an increment in testicle absolute weight in rats administered SiO2- NPs plus thymol compared to the control group, whereas it was elevated than SiO2- NPs group, although non-significant. Epididymis, seminal vesicles, and prostate gland absolute weights revealed a nonsignificant difference between all treated groups (Fig. 3A, B). Furthermore, the relative organ weight to body weight showed a nonsignificant difference between treated groups for all organs, testicles (*P* = 0.925), epididymis (*P* = 0.101), seminal vesicles (*P* = 0.719), and prostate gland (*P* = 0.458). (Figure S1)

### Sperm characteristics and the reproductive hormones

The association between the four measurements of sperm characteristics (motility, viability, abnormalities, and count) of adult male rats was tested using Principal component analysis. One component had an eigenvalue greater than 1, explaining 74.68% of the total variance. After observing the component matrix, all items in the element were retained (Table 4). However, all items are positively related except sperm abnormalities % were negatively associated with other items.

**Fig. 4:**
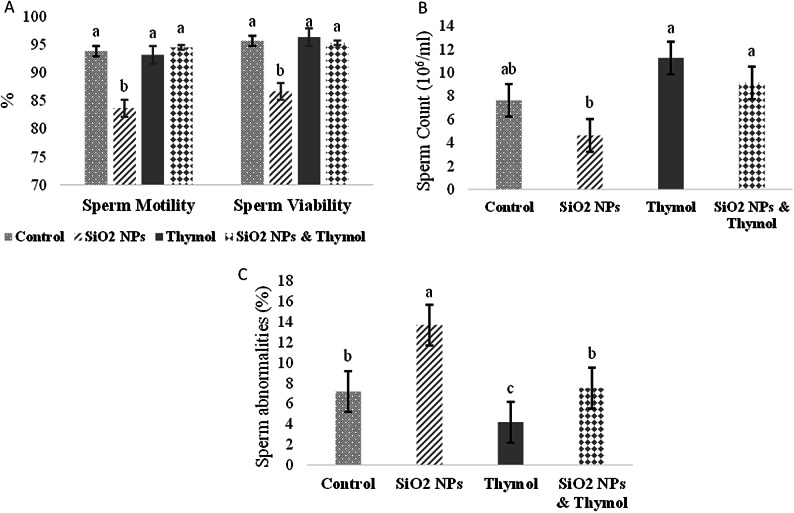
Effect of SiO₂-NPsand thymol on sperm characteristics of adult male rats: (A) sperm motility and viability (%); (B) sperm count (106/ml); (C) sperm abnormalities (%). All the values are expressed as the mean ± SEM. Different small letters(a-c) indicate significance at *p* < 0.05.

In comparison to the other groups, the SiO2-NPs group showed a markedly lower percentage of sperm motility and sperm viability (Fig. 4A). Rats given SiO2- NPs had a much lower sperm cell count than the other treatment groups (Fig. 4B). Figure 4C showed that rats given a combination of SiO2-NPs and thymol had a substantially lower percentage of sperm abnormalities than rats given SiO2-NPs alone.

**Fig. 5:**
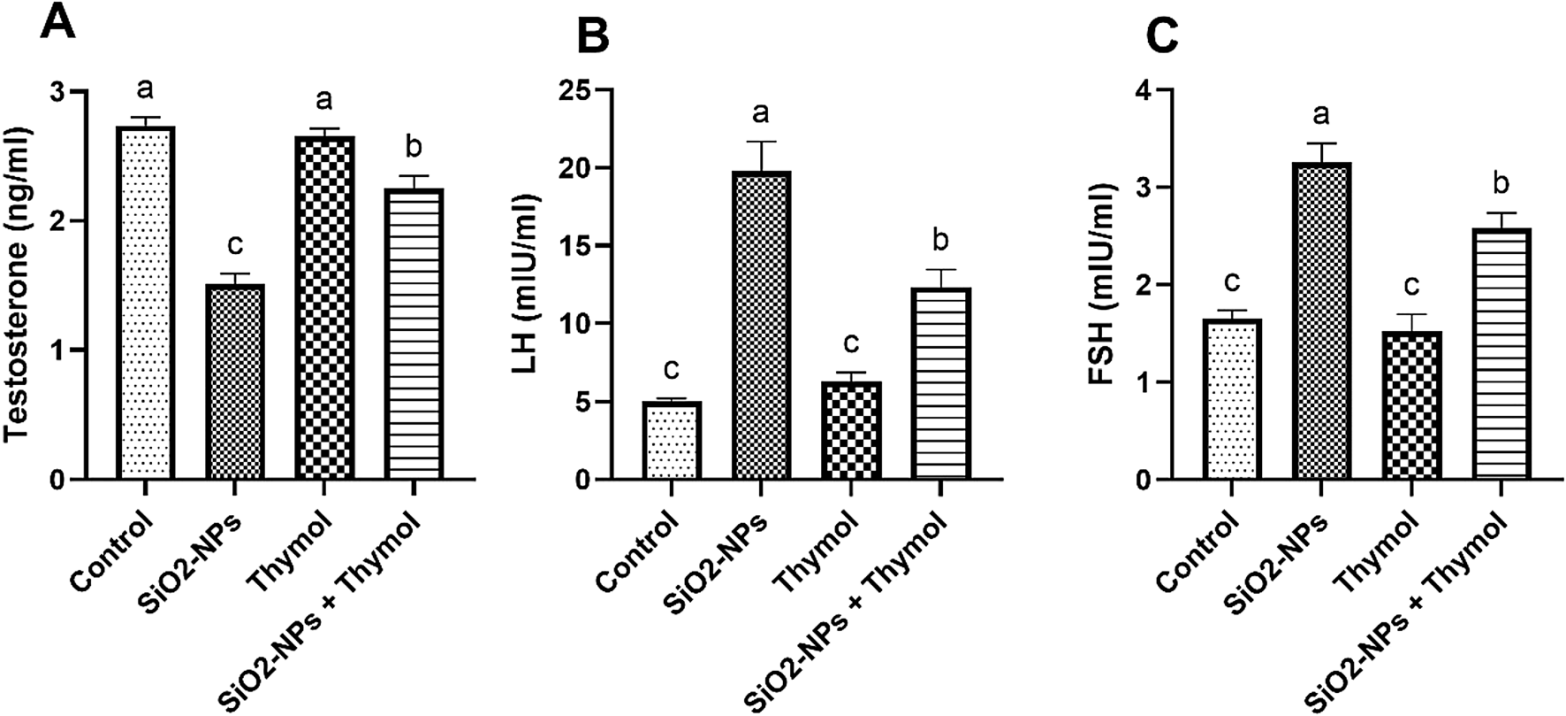
Effects of SiO₂-NPs and thymol on serum reproductive hormone levels in male rats. (A) Testosterone concentration (ng/ml). (B) Luteinizing hormone (LH) levels (mIU/ml). (C) Follicle-stimulating hormone (FSH) levels (mIU/ml). Data are presented as mean ± SEM (*n* = 6). Bars with different superscript letters (a–c) indicate statistically significant differences between groups (*p* < 0.05).

Exposure to SiO₂ nanoparticles (SiO₂-NPs) resulted in significant hormonal disruptions in male rats, as evidenced by the marked decrease in serum testosterone levels and the significant elevation of luteinizing hormone (LH) and follicle-stimulating hormone (FSH) compared to the control group (*p* < 0.05). Thymol treatment alone maintained hormone levels comparable to those of the control group, indicating no adverse endocrine effects. Notably, co-administration of thymol with SiO₂-NPs significantly mitigated these alterations, with testosterone levels increasing and LH and FSH levels decreasing relative to the SiO₂-NPs group, although not fully returning to baseline. These findings suggest that thymol offers a partial protective effect against SiO₂-NPs-induced endocrine disruption (Fig. 5).

### Histopathology

**Fig. 6:**
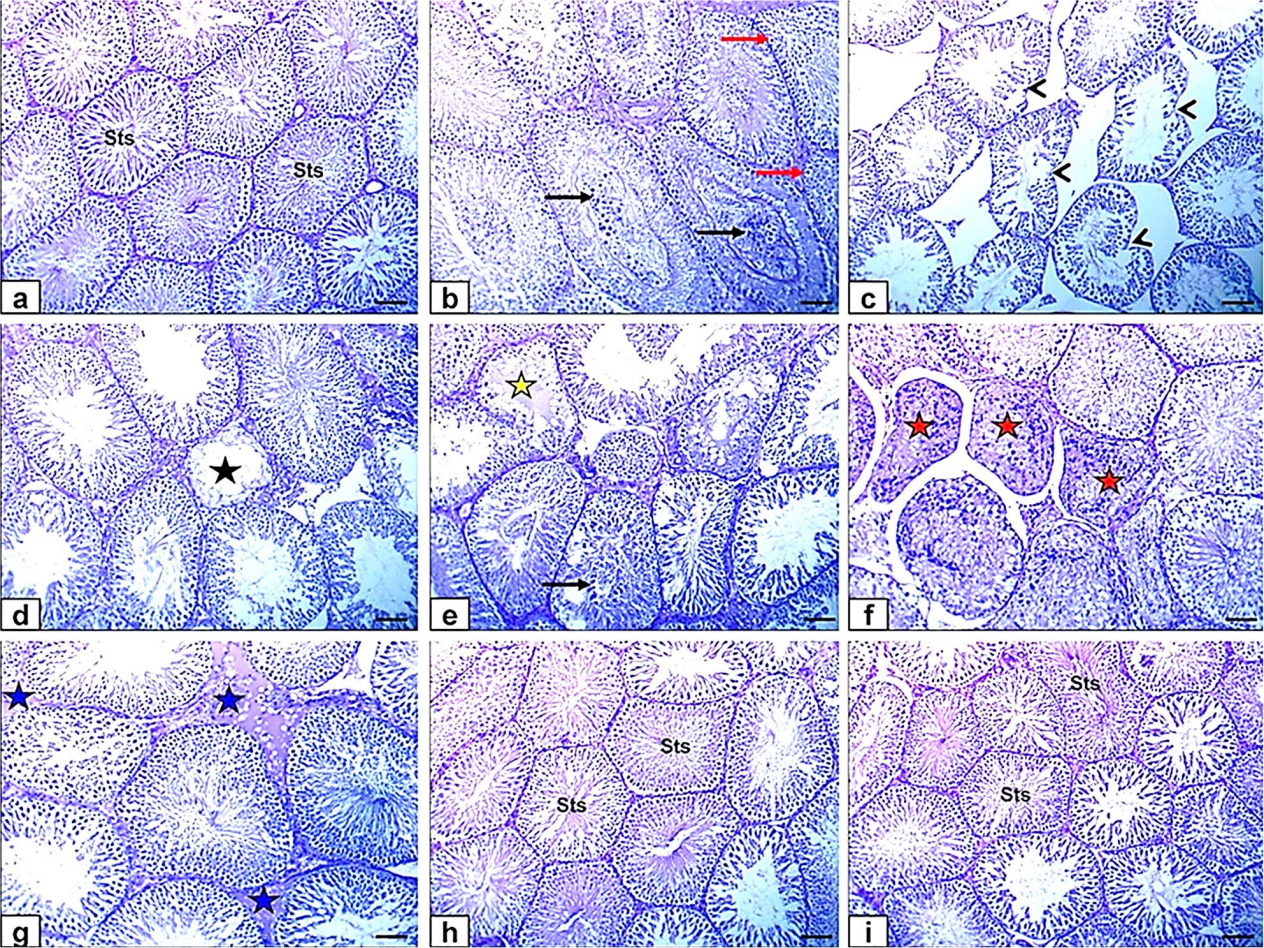
Photomicrographs of the testicle of rats stained by HE (bar = 100 μm) (a) Control group showing the normal histological structure of active mature functioning seminiferous tubules (Sts) associated with complete spermatogenic series. (b, c, d, e, f, g) SiO₂-NPs-treated group showing vacuolar degeneration of the germinal epithelium (red arrows), sloughing of the germinal epithelium into the lumen of seminiferous tubules (black arrows) beside shrunken, buckled, disorganized seminiferous tubules (black arrowheads), small-sized seminiferous tubules with no - or single - germinal cell layers (black star), hyalinization of the luminal contents (yellow star), necrotic seminiferous tubules (red stars) and faint eosinophilic albuminous interstitial edema (blue stars). (h) Thymol-treated group showing the normal histological structure of seminiferous tubules (Sts) (i) SiO₂-NPs + Thymol-treated group showing the normal histological structure of seminiferous tubules (Sts).

Testicular specimens from the control and thymol-treated groups (Fig. 6a and h) exhibited the typical anatomical structure of fully developed, functional seminiferous tubules (Sts), characterized by a complete and well-organized spermatogenic series and normal histological appearance of interstitial connective tissue and Leydig cells.

In contrast, rats treated with SiO₂-NPs displayed extensive histopathological alterations. These included severe vacuolar degeneration of the germinal epithelium, moderate sloughing of germinal cells into the lumen (Fig. 6b), and shrunken, buckled, and disorganized seminiferous tubules (Fig. 6c). Additional findings included small-sized tubules with absent or sparse germinal cell layers (Fig. 6d), hyalinization of luminal contents (Fig. 6e), and necrotic seminiferous tubules (Fig. 6f). The interstitial tissue also showed dilated and congested blood vessels and moderate, faint eosinophilic albuminous edema (Fig. 6g), indicating inflammatory damage.

Remarkably, testicular sections from the SiO₂-NPs + thymol co-treated group (Fig. 6i) showed significant histological improvement. Most seminiferous tubules regained a near-normal structure, containing elongated spermatids and spermatozoa, with only minor interstitial edema and slight vacuolization of the germinal epithelium remaining. These findings point to a notable restoration of spermatogenesis and architectural integrity.

A more detailed comparison between Fig. 6a (control) and Fig. 6i (SiO₂-NPs + thymol) reveals that although both groups display generally preserved seminiferous tubules, subtle distinctions are present. The control group shows densely packed, highly organized germinal epithelium, while the co-treated group exhibits mild disorganization and slightly reduced germ cell density in some tubules.

These observations suggest that thymol co-treatment substantially mitigated the testicular damage induced by SiO₂-NPs. While complete histological recovery was not achieved, likely due to residual nanoparticle toxicity, the improvement supports the conclusion that thymol provides a partial but significant protective effect against SiO₂-NPs-induced oxidative and inflammatory testicular injury.

For histopathological scoring, please refer to Table 5.

### Testicular oxidative stress and lipid peroxidation

**Fig. 7:**
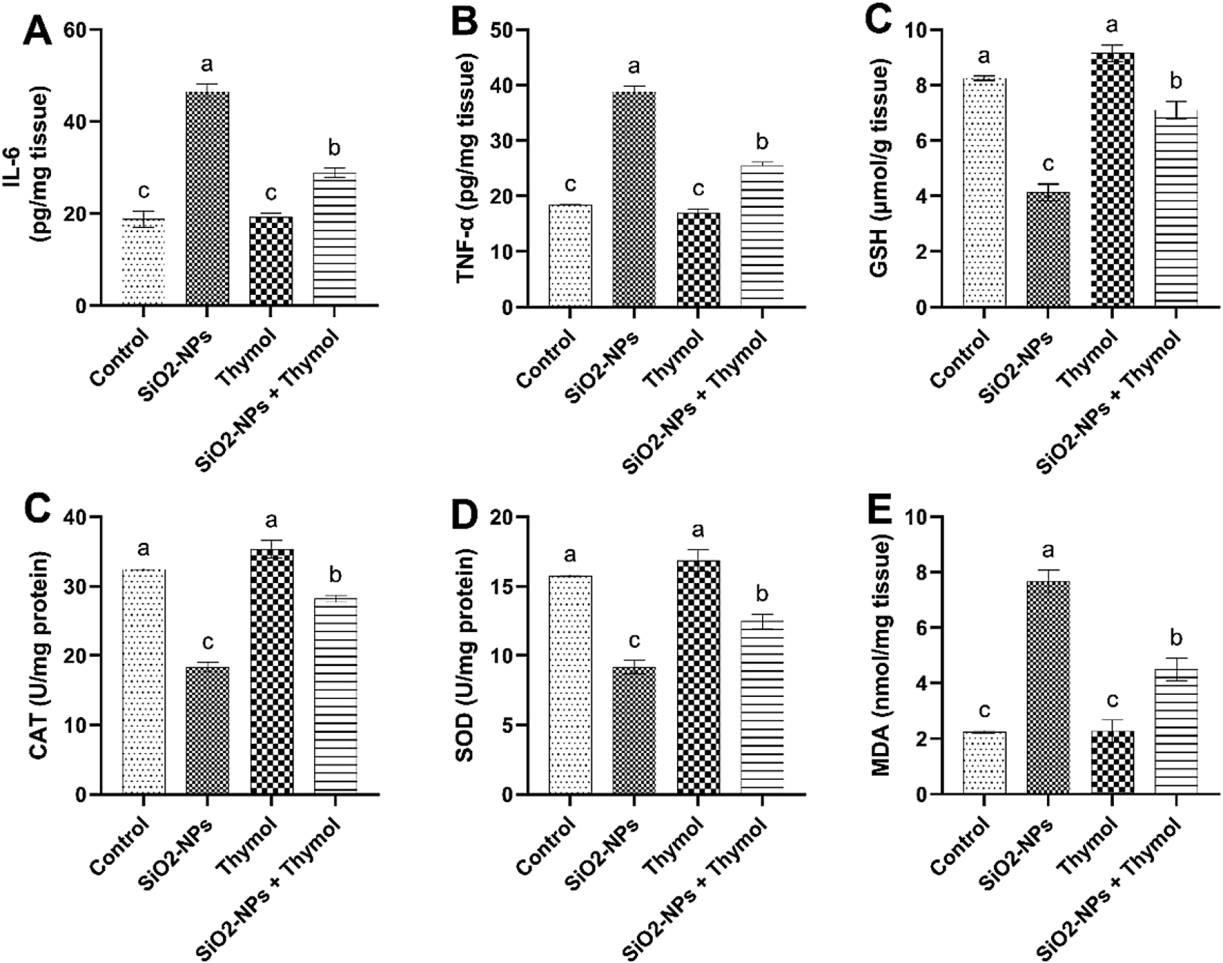
Effects of SiO₂-NPsand thymol on inflammatory cytokines, antioxidant markers, and lipid peroxidation levels in testicular tissue of male rats. (A) Interleukin-6 (IL-6) levels (pg/mg tissue). (B) Tumor necrosis factor-alpha (TNF-α) levels (pg/mg tissue). (C) Glutathione (GSH) concentration (µmol/g tissue). (D) Catalase (CAT) activity (U/mg protein). (E) Superoxide dismutase (SOD) activity (U/mg protein). (F) Malondialdehyde (MDA) levels (nmol/mg tissue). Data are presented as mean ± SEM (*n* = 6). Bars with different superscript letters (a–c) indicate statistically significant differences between groups (*p* < 0.05).

The results in Fig. 7 illustrate the effects of SiO₂-NPs and thymol on testicular tissue biomarkers linked to inflammation, oxidative injury, and lipid peroxidation in male rats. IL-6 and TNF-α levels were substantially elevated in the SiO₂-NPs group compared to the control and thymol-treated groups (*p* < 0.05). Conversely, co-administration of thymol with SiO₂-NPs led to a marked reduction in these inflammatory markers compared to the SiO₂-NPs group, though levels remained higher than in controls. Regarding oxidative injury indicators, (GSH), (CAT), and (SOD) levels were significantly decreased in the SiO₂-NPs group relative to controls, indicating compromised antioxidant defense. Thymol administration significantly enhanced these antioxidant parameters compared to the SiO₂-NPs group, suggesting a protective effect, while the combined SiO₂-NPs + thymol group showed partial restoration toward control levels. MDA, a lipid peroxidation indicator, was substantially heightened in the SiO₂-NPs group, reflecting elevated oxidative damage. Thymol treatment alone maintained MDA levels similar to control, and co-administration of thymol with SiO₂-NPs significantly reduced MDA levels compared to the SiO₂-NPs group, though not to baseline levels. These findings demonstrate that thymol mitigates SiO₂-NPs-induced oxidative injury, inflammation, and lipid peroxidation in testicular tissue.

### Gene expression

**Fig. 8:**
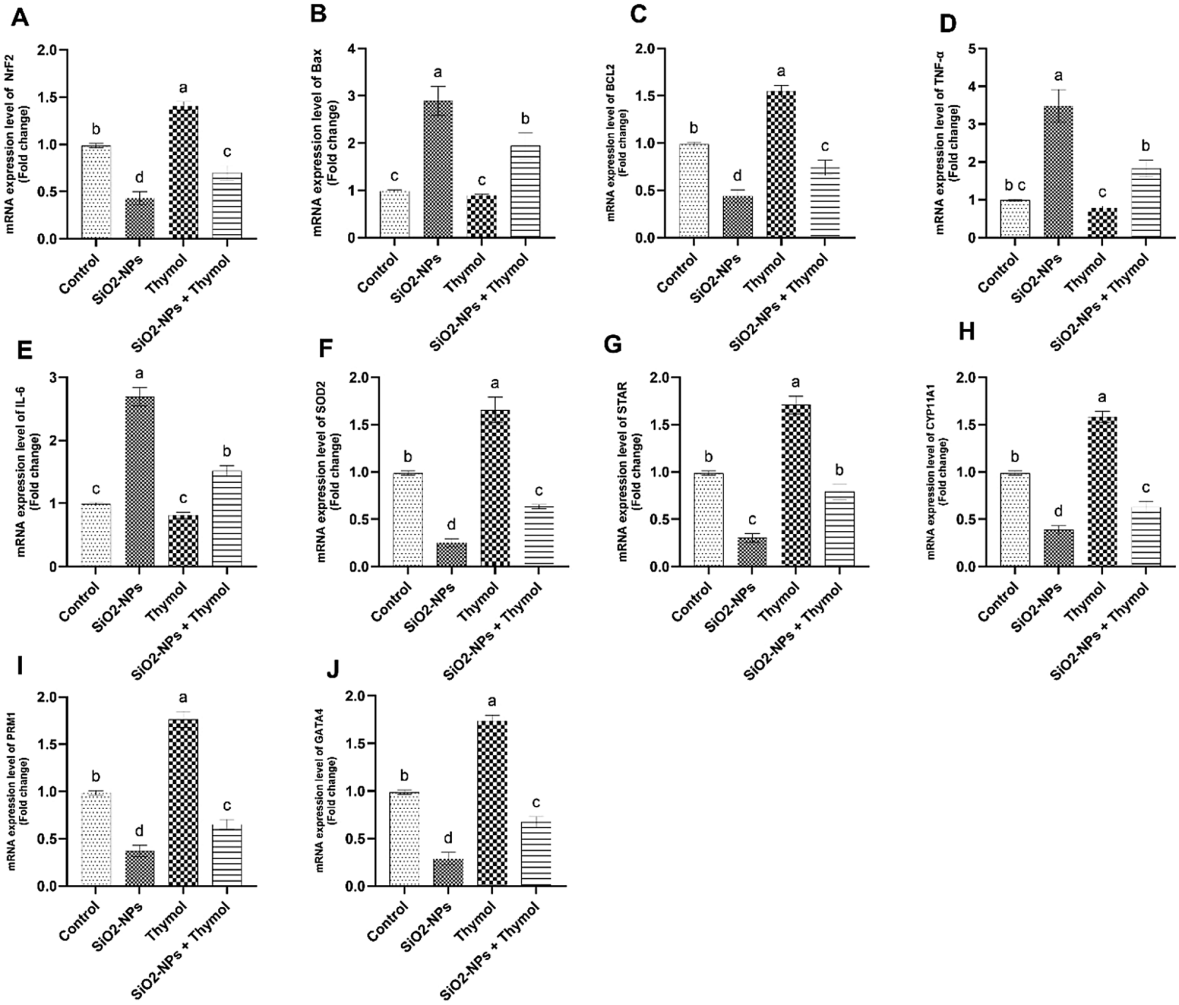
Relative mRNA expression levels (fold change) of genes involved in oxidative stress, apoptosis, inflammation, steroidogenesis, and spermatogenesis in testicular tissue of male rats. (A) NrF2 (B) Bax (C) Bcl-2 (D) TNF-α (E) IL-6 (F) SOD2 (G) STAR (H) CYP11A1 (I) PRM1(J) GATA4. Data are expressed as mean ± SEM (*n* = 6). Different superscript letters (a–d) indicate statistically significant differences between groups (*p* < 0.05).

Figure 8 presents the mRNA expression levels of critical genes involved in oxidative stress response, apoptosis, inflammation, steroidogenesis, and spermatogenesis in testicular tissues from the four experimental groups: control, SiO₂-NPs-treated, thymol-treated, and SiO₂-NPs + thymol-treated rats. Panel A showed a significant (*p* < 0.05) reduction in NRF2 expression in the SiO₂-NPs group compared to the control. In contrast, thymol treatment alone significantly (*p* < 0.05) increased NRF2 levels, and the co-administration of thymol with SiO₂-NPs partially restored NRF2 expression, showing a significant (*p* < 0.05) improvement compared to the SiO₂-NPs group. In panel B, BAX expression, a pro-apoptotic gene, was extensively (*p* < 0.05) upregulated in the SiO₂-NPs-treated group. Thymol treatment meaningfully (*p* < 0.05) reduced BAX expression, and the combined treatment group showed intermediate levels that were significantly (*p* < 0.05) different from both the SiO₂-NPs and control groups. BCL-2, an anti-apoptotic gene, had drastically (*p* < 0.05) lower expression in the SiO₂-NPs group. However, thymol administration led to a significant (*p* < 0.05) restoration of BCL-2 levels, with the co-treated group showing partial but significant (*p* < 0.05) recovery. TNF-α and IL-6 levels, markers of inflammation, were significantly (*p* < 0.05) elevated in the SiO₂-NPs-treated group. Thymol treatment, both alone and in combination with SiO₂-NPs, significantly (*p* < 0.05) reduced the expression of these inflammatory markers, indicating thymol’s notable anti-inflammatory effects. The antioxidant enzyme SOD2 (panel F) was significantly (*p* < 0.05) downregulated by SiO₂-NPs but significantly restored (*p* < 0.05) by thymol, with moderate but significant (*p* < 0.05) recovery in the combined group. Panels G and H display significant (*p* < 0.05) downregulation of STAR and CYP11A1, essential genes for steroidogenesis, in the SiO₂-NPs group, while thymol significantly improved (*p* < 0.05) their expression, suggesting restored steroidogenic capacity. Finally, spermatogenesis-related genes PRM1 and GATA4 (panels I and J) were significantly (*p* < 0.05) suppressed by SiO₂-NPs, with thymol treatment significantly (*p* < 0.05) restoring their expression. At the same time, co-treatment led to partial but significant (*p* < 0.05) normalization. These results highlight thymol’s significant (*p* < 0.05) potential in mitigating SiO₂-NPs-induced testicular gene expression disruptions.

## Discussion

With the advancement of engineered nanoparticles (ENPs), a variety of ENPs, including metal nanoparticles (NPs) like Silica nanoparticles, have found extensive applications in drug delivery, therapeutics, diagnostics, vaccines, and food products^40,41^. The widespread incorporation of NPs into daily life has drawn significant attention to their potential risks. Research has indicated that SiO₂-NPs adversely impact the male reproductive system, reducing sperm quantity and quality in rodent studies^10,12^. In the extant study, we aimed to investigate the potential defensive impacts of thymol against the SiO₂-NPs induced reproductive toxicity in male rats. The principal component analysis indicates a good association between measured parameters in sexual behavior, semen characteristics, and absolute organ weight, which are used to assess the protective efficiency of thymol against induced reproductive performance toxicity of SiO₂-NPs. Results revealed that SiO₂-NPs have a negative impact on the ability of mature male rats to reproduce as the SiO₂-NPs group showed an increase in sperm abnormalities percentage, mount latency, intromission latency, and ejaculatory latency, whereas decreased sperm motility, sperm viability, and sperm cell count. This was confirmed by histopathological examination.

These results agreed with Zhang et al.^42^, who revealed that the administration of Silica nanoparticles at the dose of (10 mg/kg BW) interferes with the sexual behavior of male rats. Furthermore, Lin et al.^43^ stated that SiO₂-NPs caused a decrease in the mating rate in male rats. The results of the semen characteristics are consistent with previous studies, which stated that SiO₂-NPs at the doses of (10 and 40 mg/kg BW) led to a reduction in sperm motility and sperm cell count and augmented sperm abnormalities in mice^13,27^. Moreover, Lin et al.^6^ deduced that silica dioxide nanoparticles by tracheal administration at the dose of 7.5 mg/kg BW every two days for 5 weeks in male rats reduced sperm count and motility while increasing sperm deformity.

In the present study, the SiO₂-NPs-treated group exhibited prolonged ejaculatory latency, a finding that, while potentially counterintuitive at first glance, aligns with toxicological impairment of sexual behavior. Ejaculatory latency is considered a behavioral biomarker of sexual desire, and longer latencies are indicative of reduced sexual motivation or arousal, which may result from neurobehavioral or endocrine disruptions^31,44^. This delay may also be associated with decreased sensitivity in achieving the ejaculation threshold, likely due to SiO₂-NPs-induced alterations in dopaminergic or serotonergic pathways, both of which are crucial for sexual reflex control^45^.

These effects are further supported by other findings in our study, including prolonged mount and intromission latencies, decreased sperm quality, and disrupted reproductive hormone levels. The co-administration of thymol significantly reversed the increase in ejaculatory latency. This ameliorative effect is likely due to thymol’s antioxidant, anti-inflammatory, and neuroprotective properties, which have been shown to restore redox balance, modulate neurotransmitter function, and improve sexual performance under toxicant exposure^19,46^. Collectively, these results reinforce the protective potential of thymol against SiO₂-NPs-induced reproductive and behavioral toxicity.

Silica nanoparticles notably elevated the levels of reactive oxygen species. Therefore, the reduction in sperm quality and number may be attributed to alterations in the redox system caused by Si-NPs^13^. Excessive ROS are thought to be harmful to sperm, as they can oxidize polyunsaturated fatty acids in the plasma membrane of sperm, damage amino acids and proteins, trigger DNA damage, and lead to apoptosis^47^. Moreover, the blood-epididymal barrier plays a vital role in maintaining the microenvironment within the duct, facilitating sperm maturation and movement, similar to the blood testicular barrier^48^. Thus, the observed effects may also be due to structural damage or dysfunction of the blood epididymal barrier induced by Silica nanoparticles^13^. Furthermore, Silica nanoparticles can negatively impact epididymal sperm quality and quantity by inducing oxidative stress and damaging mitochondrial structures, leading to disruptions in energy metabolism^9^.

On the other hand, this study showed that thymol can ameliorate reproductive toxicity of SiO₂-NPs and improve male reproductive performance as rats treated with both SiO₂-NPs and thymol exhibited a decrease in ejaculatory latency and sperm abnormalities percentage, along with increased sperm motility, viability, and sperm count in comparison with rats treated with SiO₂-NPs alone. These results are supported by Güvenç et al.^20^, who revealed that thymol (10 and 20 mg/kg BW) led to decreased sperm abnormalities and enhanced sperm motility and viability in rats. Moreover, Jafari et al.^19^ recounted that thymol at doses of (10 and 30 mg/kg body weight) improved all sperm parameters negatively affected by TiO2 nanoparticles in rats. Furthermore, Aboushouk et al.^49^ demonstrated that thymol at a dose of (100 mg/kg BW) reduced sperm abnormalities induced by methomyl toxicity in rats. Saber et al.^24^ found that co-administration of thymol at a dose of (30 mg/kg BW) in imidacloprid-intoxicated rats enhanced sperm count, motility, and viability while reducing sperm abnormalities. In addition, Tijani et al.^25^, reported that administering thymol orally at (30 mg/kg BW) improved sperm parameters that were adversely affected by hexachlorobenzene in rats. This effect could be ascribed to thymol’s ability to scavenge free radicals, reduce lipid peroxidation, and increase antioxidant activities^19,25^.

The current study demonstrated that exposure to SiO₂ nanoparticles (SiO₂-NPs) significantly disrupted the reproductive endocrine axis in male rats, as evidenced by decreased serum testosterone levels and elevated concentrations of luteinizing hormone (LH) and follicle-stimulating hormone (FSH). These hormonal alterations are consistent with previous findings in which imidacloprid (IMI), a structurally and functionally similar environmental toxicant, induced comparable endocrine disruptions^24^. The reduction in testosterone may result from direct oxidative damage to Leydig cells, which impairs steroidogenesis and reduces testosterone biosynthesis. This effect is often accompanied by a compensatory increase in LH and FSH levels, driven by negative feedback mechanisms within the hypothalamic-pituitary-gonadal (HPG) axis due to insufficient androgen signaling.

Notably, co-administration of thymol effectively mitigated these hormonal disturbances. Testosterone levels were significantly restored, while LH and FSH levels were reduced compared to the SiO₂-NPs-only group, indicating a partial normalization of endocrine function. These protective effects align with the reported antioxidant and steroidogenesis-supporting properties of thymol, which has been shown to preserve Leydig cell integrity, reduce lipid peroxidation, and upregulate steroidogenic gene expression (e.g., StAR, CYP11A1) under toxicant-induced stress conditions^19,24^. Thus, the ability of thymol to counteract SiO₂-NPs-induced hormonal imbalance likely stems from its modulation of oxidative stress and support of key molecular pathways involved in testosterone production.

The present study demonstrates the detrimental impact of SiO₂-NPs on testicular tissue, as evidenced by significant alterations in inflammatory, oxidative stress, lipid peroxidation biomarkers, and key gene expressions associated with reproductive function. The co-administration of thymol significantly mitigated these adverse effects, highlighting its therapeutic potential. SiO₂-NPs exposure led to elevated levels of pro-inflammatory cytokines IL-6 and TNF-α, indicating enhanced inflammatory responses^44^. These cytokines are critical in testicular apoptosis and impaired spermatogenesis^45^. Thymol administration significantly reduced IL-6 and TNF-α levels, underscoring its anti-inflammatory properties, likely through the suppression of NF-κB signaling. This observation aligns with previous studies that reported similar anti-inflammatory effects of thymol^24^. Furthermore, the reduction in these inflammatory markers upon thymol treatment suggests its potential role in maintaining testicular immune homeostasis.

The oxidative stress markers GSH, CAT, and SOD were significantly depleted in the SiO₂-NPs group, reflecting oxidative damage resulting from elevated ROS production^50^. This depletion compromises the antioxidant defense system, leading to lipid peroxidation, as elevated MDA level indicates. Thymol effectively restored antioxidant levels and reduced MDA concentrations, confirming its potent antioxidant activity. The phenolic hydroxyl group of thymol is likely responsible for scavenging free radicals, consistent with the findings of^20,51^. Gene expression analysis further supports these biochemical findings. The oxidative stress-responsive gene NRF2 and the mitochondrial antioxidant gene SOD2 were significantly downregulated in the SiO₂-NPs group, while thymol treatment restored their expression. This suggests thymol’s role in activating the NRF2 pathway, which is essential for combating oxidative stress^52^. Apoptosis-related genes followed a similar pattern: BAX (pro-apoptotic) expression was elevated, and BCL-2 (anti-apoptotic) expression was reduced by SiO₂-NPs, leading to cellular apoptosis. Thymol reversed these effects, indicating its anti-apoptotic potential^46^. Steroidogenesis and spermatogenesis were also affected by SiO₂-NPs, as evidenced by the downregulation of STAR and CYP11A1, essential for testosterone biosynthesis^11^.

Additionally, spermatogenesis-related genes PRM1 and GATA4 were significantly suppressed. PRM1 (Protamine 1) and GATA4 (GATA Binding Protein 4) are essential genes for spermatogenesis and male fertility. PRM1 plays a critical role in sperm chromatin condensation by replacing histones with protamines, ensuring DNA protection, genomic stability, and sperm motility^11^. Deficiencies in PRM1 expression are linked to sperm DNA fragmentation, abnormal morphology, and infertility^53^.

In contrast, GATA4 is pivotal for testicular development, regulating Sertoli and Leydig cell differentiation, testosterone synthesis through genes like STAR and CYP11A1, and germ cell survival^54^. Disruptions in GATA4 expression can lead to testicular dysgenesis and reproductive dysfunction^55^. These genes are key biomarkers and potential therapeutic targets for addressing male reproductive disorders.

While thymol has been previously investigated for its protective effects against various toxicants such as titanium dioxide (TiO₂) nanoparticles, imidacloprid, and methomyl^19,24^, the present study is among the first to comprehensively evaluate thymol’s role in counteracting reproductive toxicity induced by silica dioxide nanoparticles (SiO₂-NPs). Unlike TiO₂, which primarily induces oxidative stress through ROS generation and membrane lipid peroxidation, SiO₂-NPs have been shown to disrupt testicular architecture and function more profoundly, penetrating the blood-testis barrier, causing epithelial sloughing, germ cell loss, and inflammatory infiltration^9^.

Our findings reveal that thymol significantly mitigated these SiO₂-NPs-specific effects, restoring sperm quality, reducing inflammatory markers (IL-6, TNF-α), and normalizing key genes related to steroidogenesis and spermatogenesis (e.g., STAR, CYP11A1, PRM1, and GATA4). These outcomes suggest that thymol’s protective mechanism extends beyond general antioxidant activity and may involve targeted modulation of testicular gene expression and immune responses specific to silica nanoparticle exposure.

This study, therefore, adds novel evidence to the field by establishing thymol as a promising candidate for mitigating SiO₂-NP-induced reproductive dysfunction, distinguishing its effects from those reported in models of other toxicants.

Thymol significantly restored the expression of these genes, highlighting its role in supporting endocrine and reproductive functions. These results align with the observations of^24,25^, who reported similar results. Therefore, thymol can help protect against reproductive damage.

This study provides important insights into the protective role of thymol against SiO₂-NP-induced reproductive toxicity in male rats; however, several limitations should be acknowledged. The use of a short-term exposure model and restriction to male animals may limit the broader applicability of the findings. Future studies should explore chronic exposure, include female subjects, and adopt sex-comparative designs to provide a more comprehensive toxicological profile. While the observed protective effects of thymol are primarily attributed to its antioxidant, anti-inflammatory, and anti-apoptotic properties, the possibility of direct physicochemical interaction with SiO₂-NPs cannot be ruled out. However, it remains speculative without molecular-level evidence. Advanced approaches such as molecular docking, nanoparticle surface analyses, and binding assays are warranted to investigate this potential mechanism. Furthermore, broader transcriptomic or proteomic profiling could deepen mechanistic understanding and help identify novel molecular targets of both SiO₂-NP toxicity and thymol-mediated protection.

## Conclusion

The present study demonstrated that exposure to silica dioxide nanoparticles (SiO₂-NPs) induces significant reproductive performance toxicity in male rats, as evidenced by impaired sexual behavior, sperm characteristics, altered reproductive hormone levels, increased oxidative stress, elevated inflammatory biomarkers, disrupted gene expression related to spermatogenesis and steroidogenesis, and notable histopathological damage in testicular tissue. However, thymol administration exhibited a remarkable protective effect against these adverse outcomes. Thymol effectively restored sperm characteristics, antioxidant enzyme levels, reduced pro-inflammatory cytokines (TNF-α and IL-6), normalized the expression of critical genes (NRF2, BAX, BCL-2, STAR, CYP11A1, PRM1, and GATA4), and improved testicular histological architecture. It also significantly restored serum testosterone levels while partially normalizing elevated LH and FSH levels, suggesting endocrine system recovery. These findings suggest that thymol’s antioxidant and anti-inflammatory properties play a crucial role in mitigating SiO₂-NPs-induced reproductive performance toxicity. Consequently, thymol could be considered a potential therapeutic agent for preventing or alleviating nanoparticle-induced reproductive dysfunction. Further studies are recommended to explore the underlying molecular mechanisms and evaluate the long-term protective effects of thymol in different models of nanoparticle exposure.

**Table 1:** Primers for gene expression by RT-PCR.

Gene	Direction	Primer sequence	Accession number
*Bax*	Sense	GGCGAATTGGCGATGAACTG	NM_017059.2
	Antisense	ATGGTTCTGATCAGCTCGGG	
*Bcl-2*	Sense	GATTGTGGCCTTCTTTGAGT	NM_016993.1
	Antisense	ATAGTTCCACAAAGGCATCC	
*NrF2*	Sense	TTGTAGATGACCATGAGTCGC	NM_031789.2
	Antisense	TGTCCTGCTGTATGCTGCTT	
*TNF-α*	Sense	AACTCGAGTGACAAGCCCGTAG	NM_012675.3
	Antisense	GTACCACCAGTTGGTTGTCTTTGA	
*IL-6*	Sense	AGTTGCCTTCTTGGGACTGA	NM_012589.2
	Antisense	ACTGGTCTGTTGTGGGTGGT	
*GAPDH*	Sense	GCATCTTCTTGTGCAGTGCC	NM_017008.4
	Antisense	GATGGTGATGGGTTTCCCGT	
*SOD2*	Sense	GCTGGCCAAGGGAGATGTTA	NM_017051.2
	Antisense	TGTGATTGATATGGCCCCCG	
*STAR*	Sense	CCTGAGCAAAGCGG TGTCAT	NM_031558
	Antisense	GCAAGTGGCTGG CGAACTCTA	
*CYP11A1*	Sense	ACTTCCTGAGGGAGAACGGC	NM_017286.3
	Antisense	TCCATGTTGCCCAGCTTCTC	
*PRM1*	Sense	CGCAGCAAAAGCAGGAGCAG	NM_001002850.1
	Antisense	ACCTAAAGGTGTATGAGCGGCG	
*GATA4*	Sense	GGCTCTCTGGGAAACTGGAG	NM_144730
	Antisense	GACTGGTCTCGAACACCCTG	

Bax (Bcl-2-associated X protein); Bcl-2 (B-cell lymphoma 2); NrF2 (nuclear factor erythroid 2–related factor 2) ; TNF-α (tumor necrosis factor-alpha); IL-6 (interleukin-6); GAPDH (glyceraldehyde-3-phosphate dehydrogenase); SOD2 (superoxide dismutase 2).STAR (steroidogenic acute regulatory protein); CYP11A1 (cytochrome P450 family 11 subfamily A member 1); PRM1 (protamine 1); GATA4 (GATA binding protein 4).

**Table 2:** Loading coefficients > 0.60 for four sexual behavior items generated by principal component analysis.

Measurements	Component	
	1	2
Mount frequency		0.739
Intromission frequency	0.754	
Mount latency	0.767	
Intromission latency	0.767	

**Table 3:** Loading coefficients > 0.60 for four items of reproductive organ weight generated by principal component analysis.

Measurements	Component	
	1	2
Testes weight	0.818	
Seminal vesicles weight		0.703
Epididymis weight	0.670	
Prostate glands weight		0.617

**Table 4:** Loading coefficients > 0.60 for four sperm characteristics generated by principal component analysis.

Measurements	Component
	1
Percentage of sperm motility	0.908
Percentage of sperm viability	0.962
Sperm cell count	0.672
Percentage of sperm abnormalities	-0.886

**Table 5:** Histopathological scores for testicular lesions in all treated groups.

Groups	Control	SiO2-NPs	Thymol	SiO2-NPs + Thymol
Lesion				
Sloughing of the germinal epithelium	-	++++	-	++
Vacuolar degeneration of the germinal epithelium	++	+++++	-	++
Shrunken, buckled, disorganized seminiferous tubules.	-	++++	-	++
No - or single-germinal cell layers	-	+++	-	-
Hyalinization of the luminal contents	-	+++	-	-
Necrotic seminiferous tubules	-	+++	-	-
Interstitial edema	-	++++	-	++

The grading of testicular lesions was described as follows: (−) negative, (+) very mild, (++) mild, (+++) mild to moderate, (++++) moderate, (+++++) severe.

## Electronic supplementary material

Below is the link to the electronic supplementary material.

Supplementary Material 1

## Data Availability

The data available from the corresponding author on reasonable request.
