# Supplementary figures and images for: Thymol alleviates silica dioxide nanoparticle-induced reproductive performance toxicity via antioxidant and anti-inflammatory mechanisms in male rats
Source: Sci Rep. 2025 Jul 4;15:23913. doi: 10.1038/s41598-025-07769-x (PMC12227668; doi:10.1038/s41598-025-07769-x)

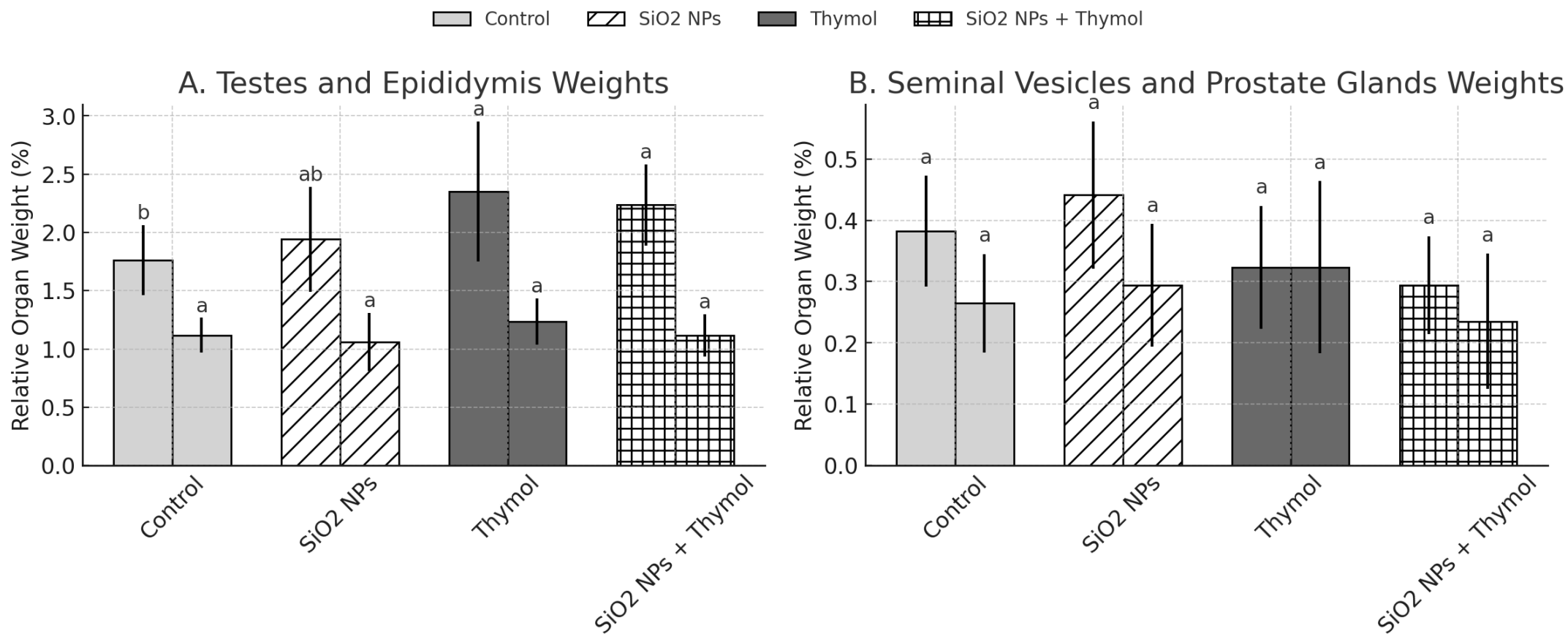

Figure S1: Relative organ weights

Supplement: Supplementary file 1 — Supplementary Material 1 [file 41598_2025_7769_MOESM1_ESM.pdf]
